# A Porro's Operation with Intraperitoneal Treatment of the Stump

**Published:** 1899-04-15

**Authors:** 


### A PORRO'S OPERATION WITH INTRA-PERITONEAL TREATMENT OF THE STUMP.

Dr. W. M. Polk,[1] of New York, advocates the performance of suprapubic hysterectomy with intra-peritoneal treatment of the stump in pregnancy at term for obstructed labour. The arguments in favour of the operation are the same as those in favour of a Porro, and are such that in his opinion it is to be preferred to Cæsarian section on the one hand and to symphysiotomy on the other, that is in conditions in which the latter operation would not bring the case within the scope of the forceps, but would only land it in the domain of embryotomy. In coming to this conclusion he is not unmindful of the fact that the condition for which the operation may have to be done is one which may be met with by medical men who are not greatly accustomed to surgical work. He says: "I would cast my vote for extirpation, and with all the greater alacrity the poorer the operator in charge of the patient. It takes more skill properly and successfully to perform symphysiotomy, eviscerate and remove a child from such a pelvis, than it does properly and successfully to deliver a living child and extirpate, from above such a pelvis." This practically agrees with what Lawson Tait said in a paper from which we quoted a few weeks ago. Dr. Polk, however, goes further and urges that "after the delivery of the child the more closely it (the operation) is modelled upon the operation of suprapubic hysterectomy for fibroids, the better," and advises that, instead of finishing the operation by leaving an extraperitoneal stump, the stump should be inverted, "after ligaturing the uterine vessels and cutting away the uterine super-structure," and, being seized from the direction of the vagina, should be drawn down, and that the bleeding should be controlled with T-shaped clamps or even ordinary hæmostatic clamps from the vaginal route. We think, however, that, appropriate as such an operation may be when performed by men accustomed to operative gynæcological work, it loses the great advantage on the strength of which it is recommended to the accoucheur in difficulties—namely, simplicity. No doubt it is a great advantage to be able to close the abdominal wall and say good-bye to the pedicle, and that the thing is feasible even in pregnancy at term may be admitted. But in an emergency operation simplicity is the great thing, and nothing can well be more simple than a Porro-Tait.

[1] Med. Rec., Mar. 4.

